# Preventing panic disorder: cost-effectiveness analysis alongside a pragmatic randomised trial

**DOI:** 10.1186/1478-7547-7-8

**Published:** 2009-04-24

**Authors:** Filip Smit, Godelief Willemse, Peter Meulenbeek, Marc Koopmanschap, Anton van Balkom, Philip Spinhoven, Pim Cuijpers

**Affiliations:** 1 Centre of Prevention and Early Intervention, Trimbos Institute (Netherlands Institute of Mental Health and Addiction), Utrecht, the Netherlands; 2 Institute of Extra-Mural Medicine, VU University Medical Centre, Amsterdam, the Netherlands; 3 Department of Research and Brief Intervention, GGNet (community mental health centre), Warnsveld, the Netherlands; 4 Institute of Medical Technology Assessment, Erasmus University Medical Centre, Rotterdam, the Netherlands; 5 Department of Psychiatry and EMGO Institute, VU University Medical Centre, Amsterdam, the Netherlands; 6 Department of Psychiatry, Leiden University Medical Centre, Leiden, the Netherlands; 7 Department of Clinical Psychology and EMGO Institute, VU University Medical Centre, Amsterdam, the Netherlands

## Abstract

**Background:**

Panic disorder affects many people, is associated with a formidable disease burden, and imposes costs on society. The annual influx of new cases of panic disorder is substantial. From the public health perspective it may therefore be a sound policy to reduce the influx of new cases, to maintain the quality of life in many people, and to avoid the economic costs associated with the full-blown disorder. For this purpose, prevention is needed. Here we present the first economic evaluation of such an intervention.

**Methods:**

Randomised trial of 117 people with panic disorder symptoms not meeting the diagnostic criteria of DSM-IV panic disorder. The interventions were time-limited cognitive-behavioural therapy *v *care-as-usual. The central clinical endpoint was DSM-IV panic disorder-free survival over 3 months. Costs were calculated from the societal perspective. Using the bootstrap method, incremental cost-effectiveness ratios were obtained, placed in 95% confidence intervals, projected on the cost-effectiveness plane, and presented as acceptability curves.

**Results:**

The median incremental cost-effectiveness ratio is €6,198 (95% CI 2,435 – 60,731) per PD-free survival gained, which has a likelihood of 75.2% of being more acceptable from a cost-effectiveness point of view than care-as-usual when a willingness-to-pay ceiling is assumed of €10,000 per PD-free survival. The most significant cost driver was therapists' time. A sensitivity analysis indicated that cost-effectiveness improves when the number of therapist hours is reduced.

**Conclusion:**

This is the first economic evaluation alongside a prevention trial in panic disorder. The small sample (n = 117) and the short time horizon of 3 months preclude firm conclusions, but our findings suggest that the intervention may be acceptable from a cost-effectiveness point of view, especially when therapist involvement can be kept minimal. Nevertheless, our results must await replication in a larger trial with longer follow-up times before we can confidently recommend implementation of the intervention on a broad scale. In the light of our findings and given the burden of panic disorder, such a new trial is well worth the effort.

**Trial registration:**

Current Controlled Trials ISRCTN33407455.

## Background

Panic disorder (PD) is characterised by a substantial influx of new cases. This influx occurs at a rate of 780 new (ie, first-ever) cases per 100,000 person-years in the adult population of 18 – 65 years in the Netherlands [1]. Their numbers should be compared with the annual number of 2,200 prevalent cases of PD in the same source population [2]. Looking at these figures one cannot escape the conclusion that 780/2,200 = 35.5% of the prevalent cases are, in fact, new cases. This casts doubts on the wisdom of relying solely on curative treatments directed at full-blown PD cases, and underscores the importance of prevention directed at sub-clinical cases, i.e. people with some PD symptoms who do not meet the diagnostic criteria for the disorder, and are thus 'at risk' of becoming cases [3]. In this context, it is worth noting that primary prevention of mental disorders appears to be a viable option [4].

Both sub-clinical and full-blown forms of panic disorder are associated with substantial costs due to excessive health care uptake, patients' out-of-pocket costs and production losses. By a conservative estimate, a full-blown PD case generates costs of about € 10,000 per patient per year, and a sub-clinical case generates € 6,000, even surpassing the costs of, for example, depressive disorder [5-8]. This suggests that offering preventive interventions for PD are likely to be cost-effective [9,10].

Against this background it was decided to develop and evaluate a brief preventive intervention based on cognitive-behavioural therapy (CBT) for panic disorder. Here, we report on the incremental cost-effectiveness ratio expressed as costs per PD-free survival time. Therapists' involvement is a significant cost driver, but can be brought under some control, for example by reducing the number of hours they invest in the intervention and by relying more on self-help on the part of the participants [11]. The latter can be done with the aid of computers [12,13]. Therefore, we conducted a sensitivity analysis for different hypothetical levels of therapist involvement: two therapists all sessions (high), one therapist all sessions (medium), one therapist only the first part of each session (low). To our knowledge this is the first economic evaluation of a preventive intervention in sub-clinical manifestations of panic disorder.

## Methods

### Design

This study was part of a larger multi-site trial. The larger trial was described in detail by Meulenbeek and colleagues [14]. In the larger trial two groups were recruited: people with relatively mild manifestations of MINI-DSM-IV panic disorder (N = 100) and people with sub-clinical manifestations not meeting the diagnostic criteria (N = 117). Here we limit attention to the latter group: people at risk of becoming cases of panic disorder. It is worth noting that the large trial was conducted with randomisation stratified for both groups. Therefore, one can consider the subset of people with sub-clinical panic disorder as constituting a trial within a trial. The study was designed as a pragmatic trial, mimicking the Dutch health care system as closely as possible in terms of patient recruitment and the methods used for intake, offering the intervention, and monitoring outcomes. Measurements were carried out at baseline (t0) and after three months (t1). In the treatment arm an extended follow-up was conducted after 6 months (t2) to monitor effect maintenance over time. The design was approved by an independent medical ethics committee (METIGG), and the trial was registered at ISRCTN under number 33407455 before commencement.

### Recruitment

**Figure 1:**
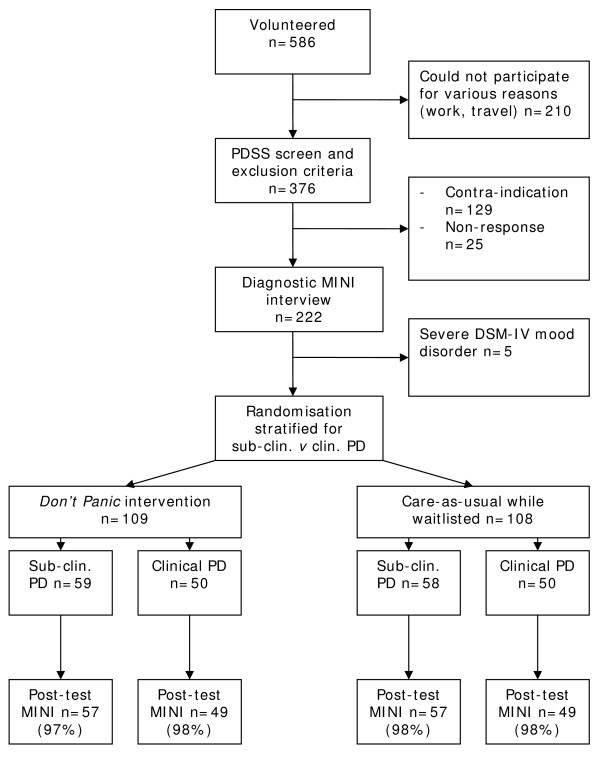
**Flow of participants through the trial**.

Participants were recruited through media announcements and via the internet. People who expressed interest underwent intake at any of the 17 participating regional mental health services. Participants had to meet the following criteria for inclusion: age over 18 years, presenting with symptoms of PD falling below the cut-off of 13 on the Panic Disorder Severity Scale (PDSS) [15], no current treatment for PD related complaints, no illness requiring immediate medical attention, and able to function independently as well as in a group. In addition, each of the candidates received a diagnostic interview with the Mini International Neuropsychiatric Interview, MINI [16]. This was done to ascertain the DSM-IV PD status [17], assess the presence of co-morbid agoraphobia, and to exclude the presence of major depressive disorder. The participants had to give written informed consent before entering the trial. See Figure 1 for the flow of participants through the trial.

### Power

With n = 139 per condition, the original trial was powered to detect a clinically relevant difference between the conditions corresponding to a mean standardised difference score (Cohen's d) of at least 0.35 in a 2-sided test at α = 0.05 and a power of (1-β) = 0.80. However, the present study is based on only a subset of the data, and is underpowered. Therefore, we refrain from hypothesis testing. Instead we present outcomes in probabilistic terms, e.g. the likelihood (in %) that the intervention is superior to care-as-usual in terms of avoiding new onsets of panic disorder in a cost-effective way.

### Randomisation

Concealed randomisation was conducted centrally by an independent third party who was 'blind' with respect to the participants. Participants were randomised in blocks of 2 with equal probability to either care-as-usual (N = 108, of these N = 58 with sub-clinical PD), or to the intervention (N = 109, of these N = 59 with sub-clinical PD). As said, randomisation was stratified for sub-clinical *v *clinical case levels of PD, and also with regard to presence *v *absence of co-occurring agoraphobia. The latter was done because it was assumed that agoraphobia is a prognostically relevant factor for outcome.

### Conditions

The experimental condition was a time-limited cognitive-behavioural preventive intervention for panic disorder: the *Don't Panic *course [18]. The choice for cognitive-behavioural therapy (CBT) was based on the understanding that CBT is the most cost-effective intervention for PD in the curative setting [19,20]. The intervention consisted of 8 sessions of 2 hours each, followed by one booster session, again of 2 hours. The booster session was offered three months after completion of the course. The course was offered by a prevention worker and a clinician to groups of 10 (9 to 12) adults. The prevention workers and clinicians were all experienced in giving CBT-based interventions. In addition, they received training in offering the *Don't Panic *course, and they were to adhere to the treatment protocol [18]. Participants received an accompanying course book [21]. The intervention was well structured and consisted of psycho-education about anxiety and panic attacks, changing life-style, managing stress, relaxation training, cognitive restructuring, interoceptive exposure and *in vivo *exposure. Each session consisted of a review of homework assignments, followed by feedback, rehearsals, information about the upcoming topic and practical skill-training. The intervention was extensively pilot tested before entering the clinical trial stage [22].

The control condition consisted of people randomised to a waiting list. They entered the waiting list on the understanding that they would receive the *Don't Panic *intervention after 3 (max. 6) months. Waitlisted participants were free to make use of health services and take medication if so required. The control condition could therefore be described as care-as-usual (CAU), but it is CAU with one exception: people were expecting to receive help for their PD symptoms in the near future.

### Outcome

The central clinical endpoint was DSM-IV panic disorder status (APA, 1994) at follow-up as measured with the Mini International Neuropsychiatric Interview, MINI [16]. In order to monitor effect maintenance over time the self-report version of Panic Disorder Severity Scale, PDSS-SR [15] was used to measure severity of panic and agoraphobic symptoms, which was done at t0, t1 and t2.

### Costs

Data on costs were collected with the Trimbos Institute and institute of Medical Technology Assessment Cost questionnaire for Psychiatry, TIC-P [23]. The TIC-P was the most frequently used heath service receipt questionnaire in the Netherlands. It used a self-report format about resource use in the last four weeks. It also contained a section on productivity losses due to absenteeism and working less efficient while at work but not feeling well. All costs that were relevant from the societal perspective were included. These costs can be grouped as follows:

• Costs of health service uptake in primary care and outpatient mental health services, including transport by ambulance, visits to emergency departments and use of ECG when tachycardia or myocardial infarction was suspected (see Table 1 for details).

**Table 1:** Direct medical and direct non-medical costs by health service type

	Direct Medical Costs (in 2003 €)		Direct Non-Medical Costs (in 2003 €)	
	
Health service type	unit	cost price ^a^	km, P, hrs ^b^	cost price ^c^
General practitioner	Visit	20.20	1.8 km, 1 h	11.10
Social worker ^d^	Contact	45.00	7 km, 3 h	28.50
Physiotherapist	Contact	22.75	1,8 km, 2 h	19.40
				
Private practice psychotherapist	Session	76.00	5 km, 2 h	19.90
Regional mental health service	Contact	124.00	10 km, 3 h	29.00
				
General Hospital – Outpatient	Visit	56.00	7 km, 3 h	28.50
Mental Hospital – Outpatient	Visit	88.00	12 km, 4 h	37.20
Teaching Hospital – Outpatient	Visit	100.00	12 km, 3 h	29.30
				
Transport by ambulance	Trip	443.00	-	-
Emergency Department	Visit	139.00	7 km, 3 h	28.50
Use of ECG ^e^	Film	36.33	7 km, 3 h	28.50
				
Home care	Hour	30.70	0 km, 0 h	0.00
Informal care (family, friends) ^f^	Hour	8.30	0 km, 0 h	0.00

^a ^Integral unit cost prices [27].

^b ^Based on average distances (in km) and travel + waiting + treatment times (in hrs) for receiving treatment [27].

^c ^Costs of 1 km = € 0.16, parking = €2.50 €, 1 h time = €8.30 [27].

^d ^From DFL 77.00 in 1993, converted into Euro, indexed for 2003 and rounded.

^e ^From  indexed by 1.10 as suggested by Oostenbrink and colleagues [27].

^f ^Valued as domestic help [27].

• Costs of medication, specifically benzodiazepines, tranquilisers and sleep medication (calculated as cost price per standard daily dose as obtained from the Pharmaceutical Compass at , plus 6% value added tax, multiplied by the number of prescription days, plus the pharmacist's dispensing costs of €6.45 per prescription).

• Patients' out-of-pocket costs for making visits to health services, i.e. costs of travel, parking and time costs (see Table 1 for details).

• Costs related to production losses due to absenteeism [24,25] and lesser efficiency while at work but not feeling well [26] were valued at €33.90 per lost hour in paid work [27] and in the domestic sphere at €8.30 per lost hour, equivalent to the cost of one hour domestic help [27].

• Intervention costs were calculated as the costs of therapists' time (€124 per hour) devoted to the intake (1 hour), 8 sessions plus one booster session (2 hours each), administration and preparation (1 hour per session), the intervention protocol (€45 per therapist), and the course book (€25 per participant). A naturalistic study showed that the number of participants in the *Don't Panic *courses was on average ten participants [22]. Accordingly, the per-participant costs of the intervention were calculated to be €750.

• Because the number of hours invested by therapists in the intervention was the single largest cost-driver, the per-patient costs for the hypothetical scenarios when therapists' involvement is reduced to 50% and 25% were calculated at €375 and €190 for both lower intensity levels, and these figures were used in the sensitivity analysis.

The costs were calculated in accordance with the pertinent Dutch guideline [27] and they reflect the full economic costs of the services. The sum of all costs is called 'total costs', and is expressed as monthly per capita costs in Euro (€). The reference year is 2003. For that year, the Organisation for Economic Co-operation and Development (OECD) equates US$1 with €0.932, taking into account both the currency exchange rate and the purchasing power in the US and in the Netherlands (see:  and look for purchasing power parities, PPPs).

### Analyses

All analyses were conducted in agreement with the intention-to-treat principle [28]. Therefore, all participants were analysed in the condition to which they were randomised, and missing endpoints at follow-up were imputed using a regression model with the best available predictors of outcome and the best predictors for dropout. The first set of predictors was required to get the most precise estimates for the missing values, the latter to correct for bias that may stem from differential loss-to-follow-up associated with t0 variables [29]. These variables were identified using logistic regression analyses with MINI PD status and dropout as the dependent variables, and age, gender, partner status, employment status were measured at baseline. With help of these variables missing endpoint were predicted and the predicted values were used to replace missing endpoints. The remainder of the analyses was conducted on the imputed data, in four steps.

First, it was ascertained how many people became a case of MINI DSM-IV PD in each of the conditions at follow-up. This was done to assess the risk of becoming a case conditional on receiving or not receiving the intervention. The probability of not becoming a PD case is equal to 1-risk, and this was interpreted as the likelihood of having a panic disorder-free survival over three months. The incremental effectiveness was computed as the difference between the probabilities of PD-free survival.

Second, the mean total costs for each of the conditions were calculated, both at baseline and follow-up. The pre-post difference in costs were computed to obtain the increase (or decrease) of costs over time within each of the conditions. The incremental cost-effectiveness ratio (ICER) was subsequently calculated as the incremental costs for a health gain of a PD-free survival over three months [30-32].

Third, a scatter plot of 2,500 bootstrapped ICERs on the cost-effectiveness plane was produced, by repeatedly drawing a random sample with replacement of size n from the original trial data (also of size n), computing the ICER and plotting that ICER on the cost-effectiveness plane [33]. This helped to produce estimates of the probability that (i) better health was generated for additional costs, (ii) that the intervention was inferior relative to the control condition because less health was produced for additional costs, (iii) that less health was generated for less costs, and (iv) that the intervention dominates because better outcomes were obtained for less costs. The bootstrap analysis also helped to obtain the median ICER and its 95% confidence interval. The latter was based on the 2.5^th ^and 97.5^th ^percentile of the distribution of the 2,500 bootstrapped ICERs.

Finally, we wanted to answer the question whether the incremental costs were balanced by the heath gains to such an extent that one would be willing to pay for the additional costs to receive the additional health gain. Making such a judgement was complicated by the fact that the exact willingness to pay (WTP) ceiling for a unit health gain is an unknown quantity. Instead of relying on a single WTP ceiling, a series of ceilings was used to calculate the probability that the intervention was more acceptable than care-as-usual from a cost-effectiveness point of view for each of the WTP ceilings. This was presented as an ICER acceptability curve [33,34], where increasing WTP ceilings were placed on the horizontal axis, while the probability of finding the intervention acceptable from a cost-effectiveness point of view was placed on the vertical axis.

All analyses were conducted in Stata [35] and Excel Professional 2003. Three analysts independently performed the analyses to cross-check results (see Authors' Contributions).

### Sensitivity analysis

The single most important cost-driver was therapists' time. It was also a cost-driver that can be brought under some amount of control, for example by relying more on (computer-aided) self-help, reducing the number of sessions, or increasing the number of participants. By way of sensitivity analyses all previous analyses were repeated under different time-investment scenarios. In the actual situation 2 therapists participated in all sessions during the full two-hour session. This is an intensive form of therapist guidance and we refer to the costs in this situation as 'high'. In an alternative hypothetical scenario a single therapist conducts the sessions, thus avoiding the costs of the co-therapist. We refer to this scenario as 'medium'. Finally, one therapist could spend one hour with the group of participants (not the full two-hours per session), which would further cut down therapist time. This scenario we call 'low'. The analyses were repeated for the three scenarios under the assumption that the effectiveness of the intervention would be reduced when therapist time was reduced. We will return to this issue later.

## Results

### Characteristics of the sample

At baseline, no differences were found between the treatment (n = 58) and care-as-usual (n = 59) conditions with regard to gender (71% female), age (mean 43.16 years, s.d. = 13.05), years of education (mean 13.72, s.d. = 3.09), partner status (75% living with a partner) and employment status (66% had a job). The mean PDSS-SR panic and agoraphobic symptom severity level in the experimental group was 6.26 (s.d. = 4.00); in the control group this was 6.01 (s.d. = 3.76), which was not statistically different (mean difference = 0.25, s.e = 0.72, t = 0.34, P = 0.732). It was also checked whether randomisation had resulted in a balanced distribution of co-morbid depression, agoraphobia and social phobia across the treatment conditions, and that this was indeed the case (χ^2^_(1) _= 0.000, P = 1.00; χ^2^_(1) _= 0.031, P = 0.86; χ^2^_(1) _= 0.006, P = 0.94, respectively). Finally, age of onset of panic disorder symptoms was also evenly distributed across the conditions (mean age of 30.6 and 31.3 years in the control and treatment conditions respectively; t = 0.34; P = 0.738).

### Adherence to the intervention

After each session the therapists registered the attendance of the participants and ascertained whether they had done their homework. On the basis of this information, 2 (3%) of the 59 subjects randomized to the experimental group did not start the course. Reasons for not starting the course were either starting another course or lack of time because of work. Forty-six (78%) subjects of the experimental group completed the course (completing the course is defined as attending at least 6 sessions). The main reasons for not completing the course were of a practical nature (e.g., work, illness). The mean number of attended sessions was 6.4 sessions. Of the attending participants, 77% had completed their homework for each session, 21% did not complete their homework for 1 session, and 2% for 4 sessions.

### Concurrent medication use

There was no significant difference in the use of medication between the groups at baseline. In the experimental group 16 (27%) participants used medication at baseline, 1 (2%) started medication during the course and 3 (5%) stopped using medication. In the control group 28 (48%) participants used medication at baseline, 2 (3%) started and 2 (3%) stopped medication in the period between baseline and T1. Therefore, it is unlikely that the present findings can be explained by changes in medication use.

### Incremental effectiveness

After the intervention, at t1, the intervention group had 51 people not meeting the DSM-IV/MINI criteria for PD. The probability of PD-free survival over three months was therefore 51/59 = 0.86. In the care-as-usual group the probability of PD-free survival was 0.74 (43/58). The incremental effectiveness was calculated as the difference between the probabilities of a beneficial outcome in each of the conditions, i.e. 0.86-0.74 = 0.12. The incremental effectiveness has been defined as the clinical parameter of interest in the remainder of this study. Its inverse equalled the number needed to be treated, NNT, as 1/0.12 = 8.3, indicating that somewhat more than 8 people with sub-clinical symptom levels have to become recipients of the intervention (rather then care-as-usual) to generate a health gain of one additional PD-free survival over three months in one of them.

### Incremental costs

Table 2 gives an overview of the monthly per capita costs (means, s.d.) in each of the conditions at t0 and t1. The base-line costs are presented in the column labelled t0. As can be seen, the total costs in the care-as-usual group (mean €346) are higher than in the treatment group (mean €222). This indicates that randomisation failed to produce evenly distributed costs across the conditions at t0. Closer inspection reveals that this difference is caused by the higher costs associated with production losses in the care-as-usual group. In the light of the difference between the conditions at baseline, we decided to calculate the pre-post differences in the costs in each of the conditions, because it would be wrong to solely focus on the costs at t1 and to ignore the 'false start' at t0. The pre-post difference of the total costs in the treatment group is €222 – €255 = -€33. Likewise the pre-post difference in the care-as-usual group is €346 – €426 = -€80. Finally, the incremental costs are calculated as the difference between the conditions, hence €80 – €33 = €47, indicating that the intervention is associated with somewhat higher monthly per capita costs than care-as-usual, but this difference is hardly appreciable from an economic point of view.

**Table 2:** Costs (in €, mean and standard deviation) by time (pre, post and difference between pre and post) and by condition (treatment, usual care) *

	PRE (t0)		POST (t1)		DIFF (t0–t1)	
	m	s.d.	m	s.d.	m	s.d.
	
TREATMENT						
• Service uptake	82	163	63	176	19	107
• Medication	9	16	8	16	<1	6
• Out of pocket	30	48	21	38	9	39
• Product. losses	102	211	163	270	-61	301
Total costs **	222	300	255	358	-33	355
						
USUAL CARE						
• Service uptake	68	83	45	60	23	86
• Medication	16	20	15	18	1	13
• Out of pocket	26	30	26	35	-1	38
• Product. losses	236	436	339	682	-103	460
Total costs	346	495	426	690	-80	483
						
INCREMENTAL TOTAL COSTS					47	847

* Numbers may not add up due to rounding. ** Costs of the intervention not included.

It is worth noting that the costs in the treatment condition were further reduced at the extended six month follow-up (t2). These costs were €222 at t0, became €255 at t1, and then became €197 (s.d. = 351) at t2.

So far we have ignored the intervention costs. These are €750 per recipient. These costs dominate the incremental costs of €47 at t1. Therefore, the intervention costs must be seen as the single most important cost driver. When the intervention costs are included in the above calculations then the incremental costs at t1 are €797, €422 and €237 for each of the (hypothetical) intensity levels.

### Incremental cost-effectiveness

Substitution of the incremental costs (€797) and the incremental effects (0.12) in the formula for the incremental costs effectiveness ratio,



indicates that the corresponding mean ICER is €6,642 per three months PD-free survival time for the factual condition. The arrhythmic mean may not provide the best estimate for the ICER. Therefore we also present the median ICER, which is based on the bootstrap distribution of 2,500 simulated ICERs. The median ICER is €6,198, which has a 95% confidence interval of 2,435 – 60,731, indicating a large amount of stochastic uncertainty.

### Sensitivity analysis

The above analyses were repeated for the hypothetical scenarios where therapist time is reduced. Reducing therapist time is associated with lower incremental costs of €797, €422 and €237, respectively for the high, medium and low intensity levels. The literature shows that reducing therapist time in delivering cognitive-behavioural therapy (CBT) for panic disorder has only a limited impact on effectiveness. To illustrate, Kenardy and colleagues [12] found that 12 sessions of CBT were only somewhat more effective than 6 sessions. Moreover, they found that 6 sessions of CBT augmented with computer-aided CBT was almost as good as 12 CBT sessions under the guidance of a therapist. This appears an interesting option for reducing therapist's time, while not sacrificing too much by way of the intervention's efficacy. Nevertheless, we prefer to make conservative assumptions and assume that the observed incremental effect of 0.12 will be reduced to 0.09 and 0.06 when therapist involvement is reduced. This results in mean ICERs of €6,642, €4,689 and €3,950 per three months PD-free survival time for the factual condition and the two hypothetical scenarios. Table 3 presents the median ICERs and their confidence intervals. The upper limit of the scenario where a single therapist is only involved in one hour per session could not be calculated, and was replaced by an infinity sign.

**Table 3:** Incremental cost effectiveness for high, medium and low levels of therapists' involvement (in € per PD-free survival over 3 months)

	High	Medium	Low
	
Incremental			
• costs	€797	€422	€237
• effects	0.12	0.09	0.06
			
Incremental cost/effect*			
• median ICER	€6,198	€3,792	€2,511
• upper bound	60,731	40,513	8
• lower bound	2,435	-39,306	-26,548

* Median ICER = 50^th ^percentile of the 2,500 bootstrap replications of the ICER

lower and upper bounds = 2.5^th ^and 95.5^th ^percentiles of the bootstrap distribution.

### Uncertainty and acceptability

**Figure 2:**
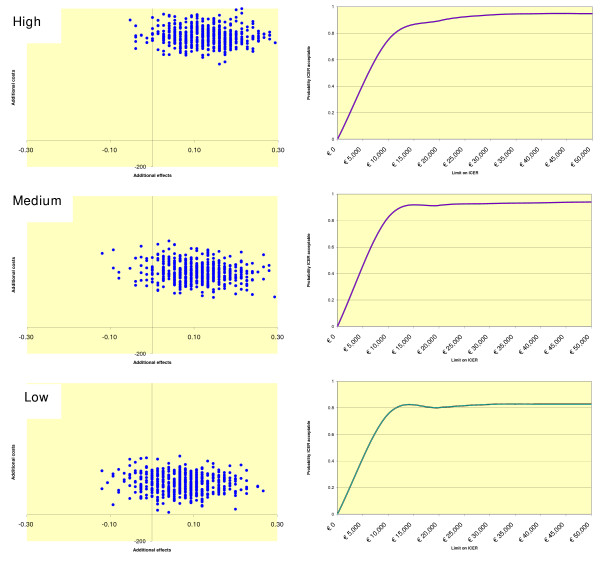
**Distribution of bootstrapped ICERs (n = 2,500) in the cost-effectiveness plane and ICER acceptability curve for each of the three intensity levels of therapists' involvement**.

A way to obtain an understanding of the uncertainty is given in Figure 2. This figure presents a scatter of 2,500 simulated bootstrap ICERs on the cost-effectiveness plane for each of the intensity levels (left-hand panel). The axes divide the cost-effectiveness plane into four quadrants. The vast majority (>87%) of the simulated ICERs fall into the north-east quadrant for each of the three intensity levels, indicating that better health is produced at additional costs by the intervention relative to the comparator condition. This is true for all three scenarios. It is also shown that the scatter shifts to the south when we move from the high to the low intensity level of therapist involvement. This indicates that the intervention gets progressively less costly in producing health gains when therapists' time is reduced. At the same time it is shown that the scatters move slightly to the west, indicating that effectiveness also diminishes when intensity levels are reduced.

The degree of uncertainty precludes statistically significant findings, but one way to tackle this problem is to adopt a probabilistic approach. That is, to present an ICER acceptability curve, which gives the probability (in %) that the treatment is more acceptable from a cost-effectiveness point of view than its alternative, given various ceilings for the willingness to pay (WTP) for a PD-free survival of three months. To illustrate, when the WTP ceiling for a PD-free period is €10,000, then the intervention has a probability of 75.2% of being more acceptable than care-as-usual under factual conditions characterised by high levels of therapist involvement. For the hypothetical medium and low levels of therapist involvement, this probability changes to 82.4% and 75.4%, respectively.

As can be seen, under the WTP ceiling of €10,000 the acceptability depends greatly on the willingness to pay: the curve is steep at the lower end of WTP ceilings, but beyond € 10,000 it becomes flat and is then fairly insensitive to WTP levels. This indicates that a decision-maker's choice at WTP € 10,000 and over is not surrounded by much uncertainty, even when the ICERs have broad confidence intervals. Below the €10,000 threshold the conclusion about the relative cost-effectiveness of the intervention depends on the precise WTP ceiling. It also depends on the intensity level of therapist involvement.

## Discussion

This study was conducted to assess the potential of a time-limited cognitive behavioural intervention for preventing onsets in panic disorder in a cost-effective way. From a larger trial the group at risk of becoming PD cases (n = 117) was selected. Of these 59 participated in the *Don't Panic *course, while 58 participants were randomised to a waiting list with unrestricted access to care-as-usual. The cost-effectiveness analysis was conducted from the societal perspective, thus including the direct medical, direct non-medical and indirect costs. The central clinical outcome was MINI/DSM-IV panic disorder (PD) free survival time over three months. Therapists' involvement was the single most important cost driver, and three intensity levels (high, medium and low) were evaluated, with the high level corresponding to the factual situation, and the medium and low levels being presented as hypothetical scenarios.

### Main findings

The data suggest that the *Don't Panic *course is more successful in preventing new onsets of DSM-IV panic disorder in people with sub-clinical symptoms than a waitlist control condition with unrestricted access to care-as-usual. The success rates are 86% in the treatment condition versus 74% in the care-as-usual condition, yielding a difference of 12% in favour of the intervention.

Not only is the incidence of a disabling disorder reduced, but this is achieved on average for €6,642 (median €6,198) per PD-free survival over three months. It can be concluded that there is a probability larger than 75% that the intervention is more acceptable from a cost-effectiveness point of view than care-as-usual when the willingness to pay (WTP) for a PD-free survival time of three months has a ceiling of €10,000. Beyond that ceiling, the likelihood that the intervention must be regarded as acceptable from the health economic point of view remains high and is not surrounded by doubt. However, below the WTP ceiling of €10,000 the acceptability of the intervention depends crucially on the WTP and is also sensitive to the level of therapist involvement. In fact, sensitivity analyses showed that the amount of therapist involvement in the intervention is an important cost driver. It is also a parameter that is under (partial) control of the management of mental health service offering the intervention. The cost-effectiveness of the intervention is much improved when therapist involvement is reduced by a factor 2 and 4. The median ICERs then become €3,792 and €2,511, even when taking into account that reduced therapist involvement may be associated with lesser efficacy of the intervention.

### Limitations

The findings of this study have to be placed in the context of its limitations.

First, the study was part of a larger trial and was not powered to detect statistically significant differences in outcomes and costs. Therefore we refrained from hypothesis testing. Instead, we took a probabilistic appraoch indicating the likelihood (in %) that the intervention is superior to care-as-usual from a health economic point of view.

Second, the time horizon of the trial was limited to only three months. This period is too short to draw convincing conclusions about preventing a disorder with an often chronic and recurrent course. Moreover, the MINI/DSM-IV PD status at t1 refers to the last month, but we interpreted it as an indicator of PD-free survival over the last three months. Hence, we may have missed an episode of PD between t0 and t1. However, it is worth mentioning that the extended follow-up in the treatment arm of the trial showed that effects are maintained for at least six months over a range of clinical outcomes (data not shown). This strengthens the impression that effects induced by the intervention persist over time, making it less likely that some people who were PD free at t1 had experienced PD between t0 and t1.

Third, the baseline costs were somewhat higher in the control group as compared with the intervention group. This difference of €124 was not significant (s.e. = 77.87; t = -1.59; P = 0.131). Nevertheless, we took care of this baseline difference in our analysis by subtracting the baseline difference from the costs at follow-up.

Fourth, in a sensitivity analysis the actual situation was compared with two hypothetical scenarios. This indicated how reducing therapist involvement would help to lower the costs of the intervention and thus improve its cost-effectiveness. Reducing therapist time could be achieved, for example, by delegating certain tasks from the therapist to a computer and offerering adjunctive computer-aided CBT for panic disorder [12,13]. However, in these scenarios we had to make assumptions of how less therapist involvement would impact on efficacy. To this end we reduced the incremental effectiveness from the observed 0.12 to hypothetical values 0.09 and 0.06, but these values are somewhat arbitrary, and the trade-offs between costs and effects may have been different from what was modelled. It is worth mentioning, however, that we made a conscious decision to make conservative assumptions about the effect of lesser therapist time on the treatment response – such that the null-hypothesis of no effect was strengthened.

In light of these limitations our findings should be interpreted with some caution.

## Conclusion

This is the first economic evaluation of a preventive intervention in panic disorder. The outcomes are encouraging, but are based on a small trial with a short follow-up. Therefore, our principal conclusion is that our findings must await replication in a larger trial with a longer follow-up before confident recommendations can be made with regard to the broader implementation of the intervention. It is our recommendation that such a trial should be conducted in the near future. After all, panic disorder is a crippling condition associated with reduced quality of life and has formidable economic ramifications for patients, the health care system, and society as a whole. To curb the massive annual influx of new cases of panic disorder, to maintain the quality of life in many, and perhaps to avoid some of the costs associated with the full-blown disorder we need a cost-effective preventive intervention for panic disorder. Our data suggest that the *Don't Panic *course is likely to be one such candidate, especially when the intervention is offered by one qualified therapist. Still, a larger replication study is needed to further test this proposition. Under the current conditions, the *Don't Panic *course is perhaps best offered as an economically affordable first step in a stepped-care approach for PD, thus allowing people to step up to more intensive forms of treatment, should that be required.

## Abbreviations

CBT: Cognitive-behavioural therapy; DSM-IV: Diagnostic and Statistical Manual, Fourth edition; ICER: Incremental cost-effectiveness ratio; MINI: Mini International Neuropsychiatric Interview; OECD: Organisation for Economic Co-operation and Development; PDSS-SR: Panic Disorder Severity Scale (self-report); PPP: Purchasing power parities; WTP: Willingness to pay.

## Competing interests

The authors declare that they have no competing interests.

## Authors' contributions

FS designed the study, conducted the analysis and wrote the manuscript. GW was principal investigator, was involved in pre-testing the intervention, was responsible for running the trial, and cross-checked the analysis of the clinical outcomes. PM designed the intervention, was involved in pre-testing it, assisted in collecting the data, and cross-checked the analysis of the economic data. MK acted as HTA advisor. PS, AvB and PC acted as a Quality Assurance Committee for this trial. All were involved in the interpretation of the data and agree with the contents of the manuscript.
